# Coronavirus disease and basic sanitation: too early to be worried?

**DOI:** 10.1590/0037-8682-0345-2020

**Published:** 2020-07-20

**Authors:** Renata Rocha da Silva, Márcio Bezerra dos Santos, Allan Dantas dos Santos, Débora dos Santos Tavares, Priscila Lima dos Santos

**Affiliations:** 1 Programa de Pós-Graduação em Ciências da Saúde, Universidade Federal de Sergipe, Aracaju, Brasil.; 2 Programa de Pós-Graduação em Biologia Parasitária, Universidade Federal de Sergipe, Aracaju, Brasil.; 3 Departamento de Morfologia, Universidade Federal de Sergipe, Aracaju, Brasil.; 4 Programa de Pós-Graduação em Enfermagem, Universidade Federal de Sergipe, Aracaju, Brasil.; 5 Departamento de Educação em Saúde, Universidade Federal de Sergipe, Lagarto, Brasil.; 6 Universidade Federal de Sergipe, Lagarto, Brasil.

**Keywords:** SARS-CoV-2, COVID-19, Water service supply, Sewage collection, Sewage treatment

## Abstract

**INTRODUCTION:**

Considering that severe acute respiratory syndrome coronavirus 2 (SARS-CoV-2) has been detected in feces, this study aimed to verify a possible relationship between basic sanitation indices and coronavirus disease (COVID-19) numbers/rates.

**METHODS:**

Data of COVID-19 cases registered in Brazil until May 28, 2020, and independent variables associated with basic sanitation were analyzed.

**RESULTS:**

A significant correlation between the number of cases and sewage treatment index/population density was observed. In addition, COVID-19 incidence and mortality rates were significantly associated with the total water service index and lethality rate was significantly associated with the sewage treatment index.

**CONCLUSIONS:**

Precarious basic sanitation infrastructure may potentially increase the SARS-CoV-2 transmission in Brazil.

The world is currently facing a novel coronavirus infection (severe acute respiratory syndrome coronavirus 2 [SARS-CoV-2]), termed as coronavirus disease (COVID-19)^1^. The initial cases emerged in Wuhan (China) in December 2019 and were associated with wild animals, especially bats. In Brazil, the first COVID-19 case was diagnosed on February 25, 2020, in the city of São Paulo. The case was of a male patient who visited Brazil from Italy, a country that was already experiencing COVID-19 cases^1^.

Clinically, COVID-19 manifestations range from mild non-specific symptoms to severe pneumonia accompanied with organ damage or failure. The most common symptoms are fever, cough, fatigue, dyspnea, myalgia, sputum production, and headache^2^. In addition, less common features include diarrhea, nausea, vomiting, and abdominal discomfort^2^. Nonetheless, most infected patients are asymptomatic and the development of severe form is associated with advanced age and chronic illnesses, such as respiratory diseases (asthma and chronic obstructive *pulmonary disease*), endocrine disorders (obesity and diabetes), and cardiovascular diseases (hypertension, cardiac arrhythmias, and cardiomyopathy). It can progress quickly and provoke respiratory distress syndrome, septic shock, metabolic acidosis, blood coagulation dysfunction, and death^3^ ^,^ ^4^. 

Coronaviruses are characterized as enveloped single-stranded RNA viruses. They are zoonotic in nature (bats are their main reservoir) and have already caused two pandemics: severe acute respiratory syndrome and Middle East respiratory syndrome^2^ ^,^ ^5^. Early cases, focused in China, were mainly caused by animal-to-human transmission; however, human-to-human transmission soon emerged through three main routes: droplet transmission (ingestion/inhalation of respiratory droplets from coughs or sneezes), contact transmission (touching a contaminated surface and subsequently touching the mouth/nose/eyes), and aerosol transmission (respiratory droplets forming aerosols in the air, which are inhaled in relatively closed environments)^2^. In the absence of an effective vaccine, various preventive measures have been proposed to curb COVID-19 transmission, including social isolation, use of facial masks, and hygiene measures, such as washing hands and cleaning surfaces and objects prior to touching/using^6^.

SARS-CoV-2 has already been detected in many biological specimens obtained from different sites of COVID-19 patients; however, the virus is primarily found in lower respiratory tract samples^7^. Notably, the virus was also detected in feces and anal and oral swabs, suggesting that SARS-CoV-2 may also be transmitted via the fecal-oral route^5^ ^,^ ^8^. As COVID-19 patients exhibit mild symptoms as in any digestive disorder, a possible hypothesis is that the virus present in the sputum is transmitted to the digestive system on swallowing^8^.

Conversely, the persistence of viable SARS-CoV-2 in water and sewage is not yet attested. A previous study, with other coronaviruses, verified a 99.9% die-off in 10 days in tap water at 23 °C and over 100 days at 4 °C. In addition, in sewage, a 99.9% die-off ranged from 2 to 3 days at 23 °C^9^. This evidence highlights the importance of basic sanitation, which, to date, is an issue in developing countries. 

In Brazil, environmental sanitation conditions are still poor. According to data from the National Survey of Basic Sanitation (2018), the country’s sewage treatment index reached 46.3% for the generated sewage and 74.5% for the collected sewage. The precarious conditions of basic sanitation also reflect lack of drinking water and minimal hygiene. 

Considering the possibility of SARS-CoV-2 fecal-oral transmission, the poor basic sanitation infrastructure, together with poor living conditions, this study aimed to verify a possible relationship between the basic sanitation indices and COVID-19 numbers/rates in Brazil and its spatial distribution.

We conducted an ecological study, using spatial analysis tools, on COVID-19 cases registered in the 27 federative units of Brazil until May 28, 2020, and assessed the correlation of COVID-19 distribution with access to water and sewage. 

Information related to cases and deaths due to COVID-19 (dependent variables) is available publicly and was obtained from the website covid.saude.gov.br (accessed on May 28, 2020), a platform updated daily by the Brazilian Ministry of Health. The mortality and incidence rates (per 100,000 inhabitants) are also available on the referred website. Furthermore, the lethality rate (%) for each federative unit/state was calculated based on the number of COVID-19 cases and deaths. The absolute number of COVID-19 cases was also evaluated as the pandemic is not yet eradicated. 

Three indices, i.e., water supply, sewage service, and treated sewage index, were used as independent variables. According to the National Sanitation Information System (SNIS; www.snis.gov.br/diagnosticos; accessed on 18th May 2020), the water supply index (IN055-%) is defined as the total urban and rural population provided with water by the service provider on the last day of the reference year (2018), the total sewage service index (IN056-%) is defined as the total urban and rural population provided with sanitary sewage by the service provider on the last day of the reference year (2018), and the treated sewage index (IN0464-%) is defined as the percentage of treated sewage, with respect to the water volume consumed. SNIS defines the treated sewage volume as the annual volume of sewage collected in the service provider’s area of operation and that has undergone treatment at the sewage treatment station (reference year, 2018). The population density data were extracted from the Brazilian Institute of Geography and Statistics (IBGE), using data from the demographic census of 2010 and the inter-census estimates of 2019^10^. 

The numbers of cases and deaths were presented in the form of absolute numbers and in form of incidence, mortality, and lethality rates. To assess the correlation between the dependent and independent variables, Spearman’s correlation test was performed, considering a 95% confidence interval. Data were considered statistically significant if *p*-value < 0.05. 

Indicators analyzed in this study were represented on thematic maps, stratified into five equal categories:

1. Incidence rate (per 100000 inhabitants): a) 0-100, b) 100-200, c)200-300, d) 300-400, and d) ≥400;
2. Mortality rate (per 100000 inhabitants): a) 0-10, b) 10-20, c)20-30, d) 30-40, and e) ≥40;
3. Water service index (%): a) 30-50, b) 50-85, and c) 85-99;
4. Sewage collection index (%): a) 0-25, b) 25-50, c) 50-75, and d) 75-85;
5. Sewage treatment index (%): a) 0-50, b) 50-90, and c) ≥90.

We used the Microsoft Office Excel 2010 software for descriptive analysis and data tabulation. The choropleth maps were designed using the QGis software, version 3.4.11 [QGIS Development Team. A free and Open Source Geographic Information System, 2020, (available from: https://www.qgis.org/pt_BR /site/about/index.html)].

Table 1 shows the absolute numbers of cases and deaths, data regarding the epidemiological indicators of SARS-CoV-2, and socioenvironmental features of each state of Brazil. The highest indicators of COVID-19 were observed in the states of São Paulo, Rio de Janeiro, Ceará, Amazonas, Pará, and Pernambuco. Although São Paulo presented the highest numbers of cases and deaths (95,865 and 6,980, respectively), Amapá and Amazonas exhibited the highest incidence (963.9 per 100000 inhabitants) and mortality (47.4 per 100000 inhabitants) rates, respectively. This was almost twice the rates observed in Ceará, the state with the fifth highest incidence and mortality rates (414.2 and 29.9 per 100000 inhabitants, respectively). However, the state of Rio de Janeiro presented with the highest lethality rate (10.8%). 

**TABLE 1:** Epidemiological indicators of severe acute respiratory syndrome coronavirus 2 and socioenvironmental information about the states of Brazil as of May 28, 2020.

Federated Unit	Cases	Deaths	Incidence	Mortality	Lethality	Total water service	Sewage collection	Sewage treatment	Population density
	(n)	(n)	rate	rate	(%)	index [2018]	index [2018]	index [2018]	[2010]
**São Paulo**	95865	6980	208,8	15,2	7,28	96.19	80.72	80.34	166.23
**Rio de Janeiro**	44886	4856	260	28,1	10,82	90.46	50.01	60.08	365.23
**Ceará**	37821	2733	414,2	29,9	7,23	58.96	40.34	90.52	56.76
**Amazonas**	36146	1964	872,1	47,4	5,43	81.14	30.36	97.35	2.23
**Pará**	33699	2704	391,7	31,4	8,02	45.62	18.36	43.70	6.07
**Pernambuco**	30713	2566	321,4	26,8	8,35	80.52	31.54	75.64	89.62
**Maranhão**	27979	887	395,5	12,5	3,17	56.39	30.45	42.81	19.81
**Bahia**	15963	570	107,3	3,8	3,57	81.62	57.29	86.80	24.82
**Espírito Santo**	12203	538	303,7	13,4	4,41	81.24	58.57	74.24	76.25
**Paraíba**	11132	318	277	7,9	2,86	74.27	48.30	88.07	66.7
**Minas Gerais**	8686	255	41	1,2	2,94	82.09	65.19	42.42	33.41
**Distrito Federal**	8300	142	275,3	4,7	1,71	99.00	85.36	100.00	444.66
**Rio Grande do Sul**	8.234	213	72,4	1,9	2,59	86.40	29.27	82.52	37.96
**Amapá**	8152	198	963,9	23,4	2,43	34.91	15.62	95.02	4.69
**Alagoas**	8055	385	241,4	11,5	4,78	74.62	17.86	81.57	112.33
**Santa Catarina**	8.000	131	111,7	1,8	1,64	89.07	28.15	98.39	65.27
**Sergipe**	6156	135	267,8	5,9	2,19	86.86	31.98	91.39	94.36
**Rio Grande do Norte**	5630	254	160,5	7,2	4,51	87.09	32.35	96.10	59.99
**Acre**	5600	122	635	13,8	2,18	47.07	18.78	99.98	4.47
**Rondônia**	4252	142	329,2	8	3,34	49.41	12.23	77.83	6.58
**Piauí**	4243	138	129,6	4,2	3,25	75.89	13.92	94.56	12.4
**Paraná**	3.984	168	34,8	1,5	4,22	94.39	73.48	99.62	52.4
**Tocantins**	3277	68	208,3	4,3	2,08	79.34	33.91	98.28	4.98
**Goiás**	3090	115	44	1,6	3,72	85.55	55.66	88.93	17.65
**Roraima**	2959	102	488,5	16,8	3,45	81.47	70.17	97.28	2.01
**Mato Grosso**	1951	51	56	1,5	2,61	89.29	42.04	87.56	3.36
**Mato Grosso do Sul**	1262	18	45,4	0,6	1,43	86.36	43.83	99.84	6.86

Considering that previous studies have already reported the presence of coronaviruses in feces^5^ ^,^ ^8^, we analyzed the correlation between epidemiological indicators of SARS-CoV-2 and the socioenvironmental features of each state of Brazil (Table 2). Interestingly, analysis of absolute data revealed a significant negative correlation between the number of cases and the sewage treatment index (ρ = -0,5269; *p*-value = 0,0047) and a significant weak positive correlation between the number of cases and population density (ρ = 0,4542; *p*-value = 0,0173). Similarly, the absolute number of deaths showed a moderate negative linear relationship with the sewage treatment index (ρ = -0,6118; *p*-value = 0,0007) and a weak positive linear relationship with population density (ρ = 0,4497; *p*-value = 0,0186).

**TABLE 2:** Correlation between epidemiological indicators of severe acute respiratory syndrome coronavirus 2 and socioenvironmental features of Brazil.

Variables	Spearman’s coefficient	***p*-value**
**Number of cases**		
Total water service index	-0,1007	0,6171
Sewage collection index	0,0635	0,7530
Sewage treatment index	**-0,5269**	**0,0047**
Population density	**0,4542**	**0,0173**
**Number of deaths**		
Total water service index	-0,1298	0,5189
Sewage collection index	0,0427	0,8324
Sewage treatment index	**-0,6118**	**0,0007**
Population density	**0,4497**	**0,0186**
**Incidence/100000 inhabitants**		
Total water service index	**-0,6319**	**0,0004**
Sewage collection index	-0,3516	0,0721
Sewage treatment index	-0,0232	0,9086
Population density	-0,2033	0,3091
**Mortality/100000 inhabitants**		
Total water service index	**-0,4641**	**0,0148**
Sewage collection index	-0,2403	0,2274
Sewage treatment index	-0,2351	0,2379
Population density	0,0064	0,9747
**Lethality (%)**		
Total water service index	-0,0656	0,7449
Sewage collection index	-0,0794	0,6938
Sewage treatment index	**-0,5284**	**0,0046**
Population density	0,2355	0,2370

The incidence and mortality rates showed a significantly negative correlation with the total water service index (ρ = -0,6319; *p*-value = 0,0004 and ρ = -0,4641; *p*-value = 0,0148, respectively). Moreover, the lethality rate presented a moderate negative relation with the sewage treatment index (ρ = -0,5284; *p*-value = 0,0046).

The spatial distribution of these variables in the north and northeast regions exhibited higher incidence than the other regions of the country (Figures 1A and B). We also observed that these states presented with lower sanitation indices (Figures 1C-F). Interestingly, the states in the northeast region that presented with better percentages of water service and sewage treatment indices (Sergipe and Rio Grande do Norte) exhibited lower COVID-19 incidence and mortality rates.

**FIGURE 1:**
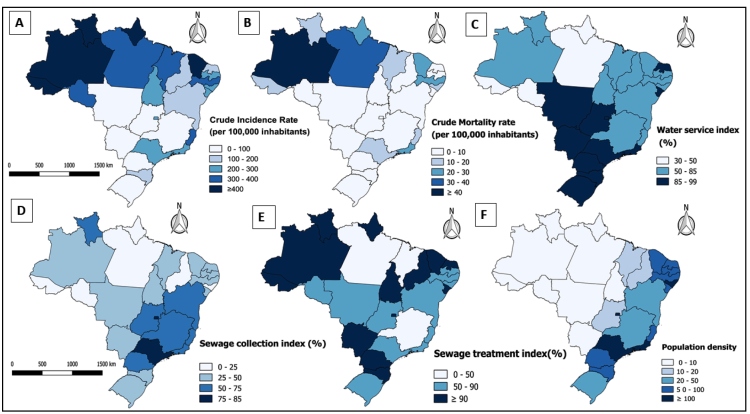
Spatial distribution of coronavirus disease and basic sanitation indices in Brazil per federative unit/state. Incidence **(A)**, Mortality **(B)**, Water service index **(C)**, Sewage collection index **(D)**, Sewage treatment index **(E),** and Population density **(F)**.

COVID-19 is an emerging infectious disease that is spreading across the country of Brazil. People living in areas with precarious conditions of basic sanitation are often more vulnerable to this disease^11^. Here, we assessed the correlation between COVID-19 numbers/rates and basic sanitation in Brazil. Although these data analyses may be premature, they highlight an ancient issue of this country.

Evidence from previous studies on SARS indicated an intestine tropism of SARS-CoV, on the basis of viral detection in biopsy specimens and stools of even the discharged patients^12^. In theory, SARS-CoV-2 could directly invade the gastrointestinal epithelium via the angiotensin-converting enzyme-2 (ACE2), as it is found on the esophageal epithelium and absorptive enterocytes of the ileum and colon, co-expressed with the TMPRSS2 prime protein. The ACE2 host cell binds to the S-protein of SARS-CoV-2 that activated by the host cell protease, transmembrane protease serine 2 (TMPRSS2)^3^ ^,^ ^12^. Moreover, the virus can be shed in feces for days even after the disappearance of respiratory symptoms^12^. Taken together, our results corroborated the hypothesis and previous findings that indicated a possible fecal-oral transmission. We found that the incidence and mortality rates of COVID-19 significantly increased with a decrease in the total water service index and the lethality rate significantly increased with a decrease in the sewage treatment index.

With the discovery of the potential of COVID-19 fecal transmission, the healthcare system and diagnostic laboratories will face a bigger challenge in regulating COVID-19 dissemination; workplace contamination should not be overlooked as well^13^. Public health education is an important measure to control COVID-19 and must be based on scientific evidence, considering its potential to prevent other infectious diseases^6^. In view of its fecal-oral transmission and other potential transmission routes, such as via drinking water, sewage, and insect vectors, further research is needed to further elaborate the effective preventive and control measures^14^.

The average rate of sewage infrastructure is 60.9% in the urban areas of Brazilian cities. In economically poor areas, such as the favelas, which are characterized as small population clusters in urban centers and rural areas (specially countryside municipalities in the northern states), these problems are more severe^15^. This is reflected in the persistent and elevated number of cases of parasitic diseases with fecal-oral transmission, such as schistosomiasis and ascariasis, which are associated with precarious basic sanitation and health education, raising potential concerns about COVID-19 transmission. These sanitation features and the presence of several population clusters in Brazil may contribute to the uncontrolled increase in COVID-19 incidence and possible reinfections. In addition, these cluster areas host 11,425,644 citizens (6% of the Brazilian population), according to the 2010 population census^10^ ^,^ ^11^. The living conditions in subnormal agglomerations are still poor when facing the COVID-19 pandemic.

Considering the basic sanitation indices presented herein and the fecal-oral route as a potential transmission approach of SARS-CoV-2, the distribution pattern of COVID-19 throughout the country needs to be monitored. However, as the pandemic continues to exist and the number of infected cases is on the rise, these correlations between basic sanitation and COVID-19 cases/deaths need to be continually assessed. More importantly, the government must direct its funds toward the improvement of basic sanitation facilities and resources, as Brazil presents with high prevalence rates of several parasitic diseases especially in regions with precarious living conditions. Hence, the question remains: Will basic sanitation become the Achilles’ heel to control COVID-19 transmission?
